# Toxicity and morbility after isolated lower limb perfusion in 242 chemo-hyperthermal treatments for cutaneous melanoma: The experience of the Tuscan Reference Centre

**DOI:** 10.1186/1756-9966-27-67

**Published:** 2008-11-12

**Authors:** Marcello Pace, Riccardo Gattai, Maria Matteini, Erminia Macera Mascitelli, Paolo Bechi

**Affiliations:** 1 Dept. of Medical and Surgical Critical Care, University of Florence, Regional Reference Centre of Tuscany for Locoregional Perfusional Therapies in Oncology, Florence, Italy; 2 Nuclear Medicine Unit, AOUC, Florence, Italy

## Abstract

**Background:**

The aim of this retrospective study was to assess the results concerning the regional and systemic toxicity and complications in 242 chemo-hyperthermal treatments (HILPs) for lower limb melanoma.

**Patients and methods:**

60 HILPs (G-A) were performed with mild HT plus L-PAM (10 mg/lt) ± D-actimomycin; 74 HILPs (G-B) with true HT (40–41.8°C) plus L-PAM (10 mg/lt) ± D-act; 108 HILPs (G-C) with true HT plus L-PAM (10 mg/lt) ± D-act plus L-PAM (5 mg/lt) additional bolus.

**Results:**

Limb toxicity was very low in G-A and in G-B; increasing toxicity (grade III = 37%) in G-C; no grade IV statistical difference was registered in all three groups, with percentage values among 1.6% and 2.7%. Systemic toxicity showed itself only in the haemopoietic parameters. No differences were registered in G-B vs G-A group. In G-C vs G-B a significative increase of systemic toxicity was seen in grade 3 (p < 0.05). Postoperative complications were acceptable. Local and systemic side-effects were transient; no permanent neurological limb deficit was registered. The postoperative mortality was recorded in 3/182 HILPs (1.6%) of the G-B and G-C groups.

**Conclusion:**

These data suggested that the technical implementations reduced the occurrence and the severity of the side effects and complications. The essential requirement for HILP is the quality assurance of the procedures. Although higher regional and systemic toxicity were observed in the G-C group caused by L-PAM additional bolus, the safeness of the procedures under the true hyperthermal regimen and the time increase of the high L-PAM concentration have assured the treatment reliability along with the increased clinical efficacy expectations of the treatments.

## Background

The isolated limb perfusion (ILP) by an extracorporeal circulation is a surgical procedure that has proven to be an effective option for the treatment of the stage III melanoma. The present ILP represents a wide treatment modality in regional metastatic cutaneous melanoma in combination with or without the regional lymphadenectomy. The rationale for ILP is based on the assumption that exposing tumours to a high concentration of citotoxic agent(s) it will increase the cell kill and can treat both macroscopic and microscopic regional disease by delivering the therapeutic effect to the whole limb, and accomplishing the change that the diffused micrometastases can be attacked in order to eliminate the residual sub-clinical disease [1-3]. The evidence of the disease progression is pointed-out in around 85.5% of the patients at high risk (thicker than 1.5 mm) with primary melanoma of the extremities developed within the locoregional distribution of the primary tumour [4]. The melphalan (L-PAM) is the most employed drug in the clinical practice. The standard of L-PAM dosage is 10 mg/lt of lower limb volume and can be administered in combination with other drugs. However, the modality and time of drug administration during the perfusion are performed in different ways from centre to centre in relation with other parameters, types and combination of citotoxic agents. During the past four decades the perfusional procedure has been refined and modified by adding heat in order to increase the tumour cell kill [5,6]. The use of hyperthermia (HT) as an antineoplastic agent is based on biological and experimental evidence [7-10]. A Different hyperthermal treatment planning has been adopted in many clinical experiences, e.g. HT levels in the range of 38 to 40°C (mild-HT) [11-13] or high temperature in the range of 40 to 42°C (borderline true HT) [14-18].

For more than twenty years, our institution has performed 242 ILPs for lower limb melanoma with L-PAM in combination with hyperthermia. The surgical technique for vascular isolation, the treatment regimens and the support apparatus technologies were modified with time, on the basis of our experimental evidence and of the obtained clinical results.

This paper analyses the results obtained with our hyperthermal ILP (HILP) procedures, in terms of toxicity and morbility, performed in three groups of patients, with different treatment schedules.

## Patients and methods

### Eligibility criteria and basic procedural guidelines

Planning the same loco-regional treatments, only patients with melanoma of the lower limb were selected for this study. Regardless of the disease stage all patients who underwent HILP were included. A computer assisted database, allowed the patients selection according to the location of the tumour and the treatment variables. Disease was staged according to the M.D. Anderson Classification System [19]. The inclusion criteria calculated, the age between 18–80 years, the performance status grade 0–1 (WHO scale), the absence of vascular diseases and other malignancies. A written informed consent was obtained.

In all patients the vascular isolation was performed at iliac level or femoral level for the second perfusion. The HILP eligibility was indicated in the multiple transit metastasis and also in only one in-transit metastasis showed up within 30–60 days after the surgical excision of the primary melanoma subsequently suggesting to perform HILP. Since this recurrence pattern represents multifocal involvement of the extremity's lymphatic system, the local excision of these in-transit lesions is frequently followed by a rapid recurrence [20]. The iliac and the obturator lymph-node dissection was performed during the vascular iliac isolation. The femoral lymph-node dissection and/or the excision of the primary tumour were performed in the same surgery procedure after HILP, when required.

The various problems and proposed solutions about the main surgical aspects regarding the vascular isolation, the fundamental criteria for an efficient heat supply and accurate and stable temperature control and measurement, have all been presented in previous works [2,18,21-23]. The review of our extensive experience was described in a recent report [24]. Before 2001, the systemic leakage was measured with RIHSA-125 based on *a posteriori *quantitative determination. [25,26] We have recently adopted the tasonermina-TNFα (Beromun^®^) protocol for real time continuous leakage monitoring during HILP, using a mobile gamma-probe and a 99mTC-Albumine micro-colloid (Albumoscint^®^) according to the Beromun ILP Procedure Guide [27].

To evaluate the post-operative regional acute toxicity effects and/or complications, a daily clinical examination of the limb is performed. Clinical observations and bio-humoral data were collected for 30 post-operative days in order to assess the adverse effects. The regional toxicity is graded according to Wieberdink et al. (table 1) [19]. During the follow-up, the occurrence of long-term sequelae were valued. The systemic toxicity was measured by WHO criteria [28]. For the results analysis (non parametric statistical test), a P-value <0.05 was considered significant.

**Table 1:** Grading of acute limb toxicity according to Wieberdink et al.

Grade I	No subjective or objective evidence of reaction
Grade II	Slight erythema and or oedema
Grade III	Considerable erythema and/or oedema with some blistering; slightly disturbed motility
Grade IV	Extensive epidermolysis and/or obvious damage to the deep tissue, causing definite functional disturbances; threatening or manifest compartiment syndromes
Grade V	Severe reaction which may necessitate amputation

### Changes in clinical practice with time

242 HILP in 213 patients (29 second perfusions) were performed in our institution from January 1984 until December 2007. The HILP treatments included three groups (table 2) with reference to the hyperthermic regimen (hyperthermic degree of limb tissue during the active phase of the treatment), the melphalan dosage with or without D-actinomycin and the period when HILP was carried out.

**Table 2:** Patients' characteristics – protocol schedules.

HILP Group		G-A	G-B	G-C
HT schedule		MILD	TRUE	TRUE
HILP = n		60	74	108
Period		1984–1989	1988–1994	1990–2007
Gender: M/F		16/44	29/45	29/79
Age: median yrs (range)		55.1 (24–76)	52 (20–70)	60.6 (30–78)
Mean body weigh: kg (range)		68.3 (45/97)	69.8 (50–96)	70 (45–138)
Mean limb volume: lt (range)		10.4 (7.5–14)	11.1 (8.2–16)	10.3 (7.5–20.5)
Stage I-II: HILP = n (%)		34 (56.7)	59 (79.8)	4 (3.7)
Stage III-IV: HILP = n (%)		26 (43.3)	15 (20.2)	104 (96.3)
HT grade		38–40°C	40–41.8°C	40–41.8°C
L-PAM	mg/lt limb vol	10	10	10
	mg for 1 lt of prime	10	10	10
	mg/lt limb vol	-	-	5 (add. bolus)
Mean L-PAM mg (range)		114.1 (85–150)	122.9 (92–195)	163 (120–240)
D-act. 1 mg: HILP = n (%)		10 (16.6)	15 (20.2)	43 (39.8)
First HILP = n (%)		55 (91.7)	70 (94.6)	88 (81.5)
Second HILP = n (%)		5 (8.3)	4 (5.4)	20 (18.5)

#### *G-A *Group

HILPs (n = 60) were performed with L-PAM (10 mg/lt of limb volume) under mild hyperthermic (38–40°C) conditions (1984–1989).

#### *G-B *Group

HILPs (n = 74) were performed with L-PAM (10 mg/lt) under borderline true hyperthermic (40–41.8°C) conditions (1988–1994).

#### *G-C *Group

HILPs (n = 108) were performed with double L-PAM bolus (10 mg/lt and additional bolus of 5 mg/lt) under borderline true hyperthermic (40–41.8°C) conditions (1990–2007).

In the G-A Group, the administering of L-PAM was performed when the limb temperatures reached range 38–39°C; in the G-B Group at 39°C and in the G-C Group, when the limb temperatures reached 39°C in 53/108 HILPs and 40°C in 55/108 HILPs. In all treatments the standard dose of L-PAM 10 mg/l was administered as a single bolus in the arterial line. In the G-C group, the additional L-PAM bolus was administered 30 minutes after the first L-PAM bolus. The active phase of treatment was maintained for 60 minutes; only in 31 HILPs (according to WHO trial n°17 protocol) [29] the active phase was maintained for 90 minutes. D-actinomycin (1 mg standard dosage) was administered before L-PAM only in 10 HILPs (16.7%) in the G-A group; 15 (20.2%) in the G-B; 43 HILPs (39.8%) in the G-C.

### HILP Procedure

The technique and methodology used for HILP, were previously described. [2,23,30]. Hereinafter, we report the currently procedure that we have adopted, resulted from the outcome of our clinical experience in borderline true HT treatments with a particular reference to the adopted advices in preventing the regional and systemic adverse effects. In the lower limb HILP, we perform the deep vascular isolation at iliac level after the external iliac and obturator lymphnodes dissection. Under systemic heparization (150 UI/Kg b.w.) the iliac vessels are cannulated and the extracorporeal circuit is inserted. Optimal vascular blockage is obtained by the temporary clamping of the internal iliac artery, the common iliac vein and by the legation of the inferior epigastric and the circumflex vessels. For the femoral cannulation, the technical details about this vascular approach were previously reported [2]. The cannulas, usually 16F for the artery and 24F for the vein, guarantee high perfusate flow rate in order to reach the appointed hyperthermal temperature of the limb tissue. The surface isolation for iliac and femoral perfusion is obtained by the constriction of the belt fastened to a pair of (modified) Steinmann nails inserted into the subcutaneous layer of the lower abdominal wall (*abdominal tourniquet*) [26].

The monitoring of the limb temperature is secured by six probes inserted in the cutaneous, subcutaneous and muscular tissues: three probes are positioned every time at the middle third of the thigh and the others three at the middle of the leg. The limb is wrapped by a warm water circulation blanket that in association with the heated perfused, allows to obtain homogeneous tissue temperatures throughout the limb and to reach the operative thermal range without the need of heating the perfusate over 42°C at the oxygenator. Consequently, strict and mandatory requirements arise in the borderline true HT and a temperature close to the physiological tolerability limit (41.5–41.8°C at the arterial cannula) is required. This safety limits make an accurate and real-time control of the temperature inside the various tissues and compartments of the limb. We have lately used a new device (Meditherm II, Gaymar, AB Medica) of the external heat source in which the water blanket temperature is set at 41°C for the whole time of the surgical procedure and during the perfusional treatment until the washing-out starts. Care is taken to guarantee the thermal limb isolation in order to reduce the heat loss [30].

During the time of the surgical vascular isolation, the extracorporeal circuit is primed with plasma expander (1000 ml with sodium heparin 500 UI) and circulation is started with a short circuit between the arterial and venous lines. The prime pre-heating is thus obtained (40°C). The prime volume expressed in liters, is added to the total L-PAM dosage. Before the administering of L-PAM, the systemic leakage is always monitored.

**Figure 1:**
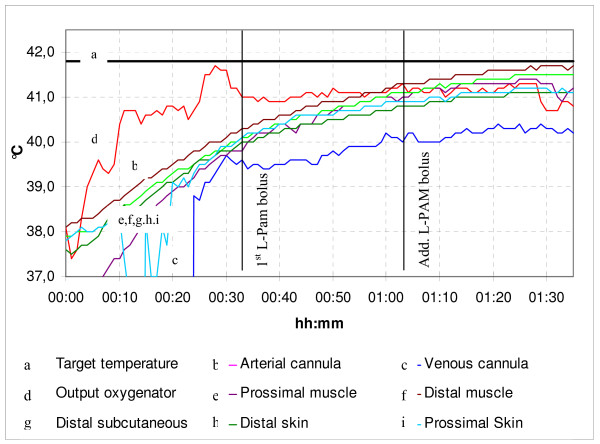
D.A. – ILP (28.03.07) – Typical temperature profile during lower limb perfusion with L-PAM bolus (10 mg/lt) plus additional bolus (5 mg/lt).

**Figure 2:**
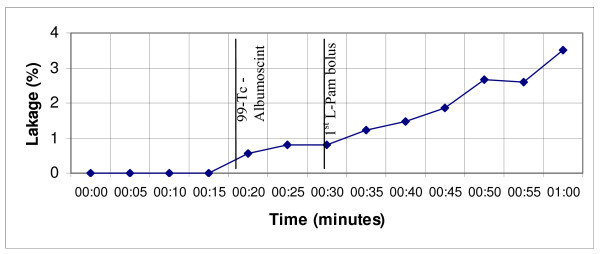
D.A. – ILP (28.03.07) – Continuous leakage monitoring by 99mTc- Albumoscint and by manual gamma probe (leakage/time profile).

When the extracorporeal circulation starts, the administration of L-PAM is performed when the limb temperature has reached 40°C and the active phase starts. D-actinomycin when employed, is administered ten minutes before L-PAM. The chemo-hyperthermal phase is maintained for 60 minutes at the temperature range of 41–41.8°C. The additional bolus (5 mg/lt) is administered 30 minutes after the first L-PAM standard dosage, during the active phase, in order to maintain a high concentration for a longer time. The perfusate flow is maintained at 600–800 ml/min during the transitory thermal phase of the prime heating. Typically, when the limb temperature reaches 40°C, the flow rate may be reduced up to 300–400 ml/min before the drug administering. This action allows to contain the systemic leakage and at the same time checking that the various temperatures remain substantially constant in time. Afterwards, the perfusate flow rate is again risen step by step so that the temperature will rise to 41.5–41.8°C. Figs. 1 and 2 show the temperatures/time and leakage/time typical profiles of the borderline true HILP with double L-PAM bolus.

At the end of the active phase the washing-out is performed. The perfusate is drained out and at the same time the circuit is rinsed with 6000 ml of electrolyte solution at 38°C and consecutively 1000 ml of hydroxyethilic starch (Amidolite^®^) solution. After the washing-out completion, the venous line is clamped and 1000 ml of hydroxyethilic starch are perfused into the arterial line for protection against oedema and platelet aggregation. The tourniquet is released and the vascular cannulas are removed. A tubular drainage is placed in the retroperitoneal seat for 3–4 days. When the femoral lymph-node dissection is performed, two vacuum-scaled Redon drainages are placed at the femoral seat for 8–10 days. No fasciotomies are performed. Heparinization is not reversed and patients are maintained under heparinic control during the postoperative course until they have recovered full mobility.

## Results

A total of 242 HILPs (213 pts) were selected for this study: 168 women (69.4%) and 74 men (30.6%) with a mean age of 56.6 years (range 20–78). The main purpose of this study was to validate the feasibility and security of more intensive perfusional treatments with hyperthermal regimen, close to the upper limit of physiological tolerability. In this paper the toxicity and the complications checked in the three HILP-groups performed in three periods of our clinical experience were analysed and compared.

Two essential requirements were reached: (1) the thermic profile of the soft tissues of the entire limb was always maintained during the active phase of the treatment [24] at a constant temperature between 40 and 41.8°C; (2) the systemic leakage data obtained from the two used procedures, have guaranteed values at 5 minutes constantly below the threshold value of 5% [26].

In the HILPs collected rate, the disease stage was reported in table 3: in G-A stage I (48.4%) was as part of the EORTC Trial n°15 [31] and in G-B stage I (78.3%) was as part of the WHO Melanoma Programme Trial n°17 [29]. In stage IV the HILPs were palliative treatments.

**Table 3:** HILPs (%) collected according to stage disease

HILP Group	Stage			
	
	I	II	III	IV
G-A (n = 60)	48.4	8.3	43.3	-
G-B (n = 74)	78.3	1.3	20.4	-
G-C (n = 108)	0.9	2.7	92.7	3.7

The grading of the acute limb toxicity in the single HILPs group is reported in table 4. The grade II and III reactions were observed with no different rate in G-A and G-B; whereas the G-C group presented a higher toxic reaction (grade III in the 37% of HILPs).

**Table 4:** Regional toxicity grade according to HILP group (%)

HILP Group	Grade (acc. to Wieberdink et al)				
	
	I	II	III	IV	V
G-A (n = 60)	1.6	85.2	11.6	1.6	-
G-B (n = 74)	2.7	81.1	13.5	2.7	-
G-C (n = 108)	4.6	56.6*	37*	1.8	-

* G-B vs G-C (p < 0.005)

In the groups G-B and G-C (182 HILPs) in which the borderline true hyperthermia was used, the regional acute toxicity was observed with grade II in 121 HILPs (66.4%); III in 50 (27.5%); and grade IV in 4 (2.1%).

Although the little number of second perfusions, the regional toxicity occurred with more severe compared to the first HILP. G-A: grade II in all HILPs; G-B: 75% vs 83% grade II and 25% vs 13% grade III; G-C: 50% vs 57% grade II, 45% vs 35% grade III and 5% vs 2% grade IV.

The clinical parameters observed in the grade II reaction were transient (7–10 days), the erythema and oedema slide were often not combined. In HILPs with grade III more intense erythema and/or oedema occurred, especially when a regional node dissection was also performed. Some superficial and confined blistering seldom occurred with the *restitution ad integrum *within 5–8 days. Only one case (grade III G-C) was associated to severe pancitopenia with acute respiratory distress. The functional morbidity or nerve effects occurred in one HILP (Trial 17-G-B group) with grade II toxicity and with persisting cutaneous limb hyperaesthesia. In one HILP-grade III (G-C) it was observed a continuous and permanent diffused pain to the entire limb and no response to the pharmacological treatment. The Grade IV reaction was in two cases: in one HILP G-B (Trial n°17) the reaction was observed with wide areas of deep burn in the cutaneous and subcutaneous tissue of the thigh and leg, and plastic surgery was needed; the next one (G-C) suffered a serious blistering of the thigh from transitory to moderate reduction of the motility function. Two other IV grade cases' reaction with neuropathy respectively occurred in G-B and G-C; each case developed a multi-organ failure (see table 5).

**Table 5:** Acute systemic toxicity (HILP %)

HILP Group	G-A (n = 60)					G-B (n = 74)					G-C (n = 108)				
Grade	0	1	2	3	4	0	1	2	3	4	0	1	2	3	4

RBC	3,3	26,8	56,6	13,3	0,0	2,7	21,6	54,0	17,6	4,1*	0,9	15,7	50,0	27,8^•^	5,6
Hb	3,3	16,7	63,3	15,0	1,4	2,7	16,2	50,0	25,7	5,4	2,8	8,3	39,9^•^	41,6	7,4
WBC	18,3	11,7	31,7	25,0	13,3	36,5*	10,9	12,1*	24,3	16,2	10,2^•^	10,2	20,4	26,8	32,4^•^
PLT	61,7	21,7	10,0	5,0	1,6	71,6	9,5*	8,1	4,0	6,8*	23,1^•^	31,7^•^	12,9	12,0^•^	20,3^•^
ALT/AST	30,0	48,3	15,0	5,0	1,7	48,7*	33,8	13,5	4,0	0,0	57,4	29,6	7,4^•^	2,8	2,8
CPK	15,0	30,0	28,4	18,3	8,3	29,7*	21,7	27,0	13,5	8,1	37,9	24,1	24,1	8,4	5,5
Creatin	76,8	20,0	1,6	1,6	0,0	87,9	9,5*	1,3	1,3	0,0	91,8	5,5	0,9	0,9	0,9

*G-B vs G-A statistical difference is significant; ^• ^G-C vs G-B statistical difference is significant

The acute systemic toxicity was evaluated according to the WHO criteria [32]: blood analysis was performed when required according to the clinical conditions, at least every three days for the first post-operative period (10 days) and to the completion of 30 days. Table 6 reports the most frequent haematological parameters that in HILP treatments are liable to be changed. The analysis of the collected data pointed out that in G-A and G-B the most significant haematological data do not differ. In particular, in both groups the RBC and Hb parameters in main grade 2 and 3 of toxicity were collected. The WBC and PLT parameters in main 2 and 4 grade. Whereas a remarkable rate of haematological parameters tested in the G-C treatment were collected in main grades 2, 3 and 4. These remarks showed a transitory increase of the haematological and medullar toxicity with a homogeneous supply in the high-grades. No major toxicity was recorded from the analysis of the hepatic and renal function in the three groups. The systemic toxicity in all the treatment groups was typically observed within the five post-operative days, followed by early normalization (5–7 days). In some cases the need to administer some red blood cell units during early post-operative days.

**Table 6:** Post-operative regional and systemic complications in HILP groups.

HILP Group	G-A n (%)	G-B n (%)	G-C n (%)
Lymphocele	4 (6.6)	7 (9.4)	19 (17.5)
Bleeding necessitating surgery	-	1(1.3)	3(2.7)
Deep venous thrombosis	-	2(2.6)	3(2.7)
Pain	1(1.6)	1(1.3)	1(0.9)
Peripheral nerve palsy	-	1(1.3)	1(0.9)
ARDS	1(1.6)	-	1(0.9)
Pleural effusion	-	1(1.3)	-
Pulmonary microembolism	-	1(1.3)	-
Death (MOF or ARDS)	-	1(1.3)	2(1,8)

The clinical observation, reported in table 5, evaluated the side-effects and the complications attributable to the complex treatment, correlated with the surgical techniques combined with the citotoxic therapy, The loco-regional complications were more frequent in G-B and G-C groups. Following the drains, after 8–10 days the development of a lymphocele was observed in four HILPs of G-A group (6.6%); in G-B: 9.4% and in G-C: 17.5%. This complication was treated with punctures or repeated placement of a suction drain during the follow-up. Retroperitoneal bleeding in the size of the iliac vascular isolation occurred in four HILPs and required the wound revision. A homolateral deep venous thrombosis was recorded in five HILPs with complete recovery through a standard medical therapy. An acute respiratory distress was observed in two HILPs, they were transient and showed a favourable clinical evolution by reducing the blood dilution after the washing-out. One bilateral pleural effusion and a pulmonary microembolism were seen in two HIILPs (G-B – Trial n°17); both situations were resolved with a sub-intensive care within 7–10 days. Post-operative mortality occurred in 3/242 HILPs (1.2%). One case (G-B) with MOF caused by a compartmental syndrome, died on the6^th ^post-operative day. The other one (G-C) died 10 days after HILP, suffering from severe and early bone marrow followed by a septic shock. Severe ARDS in one HILP (G-C) with grade III reaction caused death after 20 days.

## Discussion

Higher-temperature treatments are associated in the published results with greater efficacy but also with major toxic effects especially when the contribution of the associated cytostatic drugs is considered [28,33]. In our institution, over the years, progresses in technologies and methodology have improved the results with regard to side-effects through the carrying out of safer and more acceptable procedures in spite of the use of the borderline true hyperthermia. In addition, previous clinical results reported in our pilot study [34] demonstrated that borderline hyperthermia with an increase of L-PAM concentration in the advanced lower limb melanoma (stage III) has improved the locoregional disease-free interval and the overall survival rate.

In this report the analysis of the acute regional toxicity data in HILPs-GA (mild HT) allows to remark that only a moderate toxicity was observed with grades II/III (85/11%). The Wieberdink grades of the HILPs-GB (true HT) compared with the data of GA group with the same standard L-PAM dosage, confirm that the use of true hyperthermia up to 41–41.8°C do not cause a higher regional toxicity. In our experience with true hyperthermal treatments, acceptable regional toxic reactions can be achieved on condition that the high value of temperature, close to the maximum physiologically tolerable limit, should never be higher than 42°C at both levels of the limb tissue and of the perfusate during a long treatment time [24,35,36]. In addition, the essential requirement was a critical control of the temperature through the limb-circuit system which intrinsically implies uniformity in space and time [23]. Comparing the Wieberdink grade between G-C (true HT plus double L-PAM bolus and G-B (true HT with standard L-PAM dose) pointed out a higher increase of grade III vs grade II in G-C group. These data show that the increase of toxic reactions is due to the high L-PAM administered concentration. Nevertheless, in both groups (true HT) compared with G-A, an increase of the grade IV toxicity was not registered, and the toxic reactions in the HILPs group (true HT) were included in II-III Wieberdink grades. These remarks, especially for grade III were valued, in our experience, as a moderate toxicity considering that the clinical events were often single and/or transitory.

The acute systemic toxicity regarding the haematologic parameters has pointed out a progressive increase towards higher toxic grades (3–4) in G-C vs G-B group, whereas no statistical difference was matched between G-B and G-A groups. These results induce us to make the following remarks: (i) the vascular isolation technique use in our procedure has not influenced significantly the drug systemic leakage, on condition that continuous and strict leakage monitoring is adopted; (ii) in the treatments with true-HT (74 HILPs) the increase of the systemic toxicity has not occurred in comparison to mild-HT treatments; (iii) a higher toxicity found in G-C vs G-B group with the same HT conditions, must be attributed to the high L-PAM concentration (additional bolus); (iiii) high L-PAM concentration over along time is liable to the damage of the haematopoietic function, whereas does not directly interfere with the hepatic and renal functions.

The postoperative regional and systemic complications in all HILP groups have not showed a statistical difference; however, a trend on a moderate increase was matched in G-C group. These remarks were seen to be in relation with the higher median age of the patients with possible comorbidities and/or advanced disease (stage IIIAB) necessitating more invasive combined surgery (i.e. simultaneous regional lymph-node dissection) as occurred in G-C group.

In our opinion according to Knorr et al [37] the overall side-effects and complications data found with these HILP procedures are acceptable in the face of the more intensive perfusional treatments. We have reason to believe that the analysis of our clinical data involving borderline hyperthermia with or without increase of the L-PAM concentration, will confirm the benefit of our innovative perfusional procedure by improving the local control of the disease and possibly the survival rate.

## Competing interests

The Authors have no direct nor indirect financial and no-financial interests in the procedures and techniques presented in this paper.

## Authors' contributions

All the Authors have made substantial contributions to conception, design, acquisition data, analysis and interpretation of data. MP was involved in the conception, design, analysis and interpretation of data. RG was involved in the design, acquisition of data, analysis and interpretation of data. MM and EMM were involved in the acquisition of data and the analysis of data. PB was involved in the conception, design, analysis and interpretation of data.
